# MHC Class II is Induced by IFNγ and Follows Three Distinct Patterns of Expression in Colorectal Cancer Organoids

**DOI:** 10.1158/2767-9764.CRC-23-0091

**Published:** 2023-08-09

**Authors:** Oliver J. Pickles, Kasun Wanigasooriya, Anetta Ptasinska, Akshay J. Patel, Helen L. Robbins, Claire Bryer, Celina M. Whalley, Louise Tee, Neeraj Lal, Claudia M.A. Pinna, Nahla Elzefzafy, Philippe Taniere, Andrew D. Beggs, Gary M. Middleton

**Affiliations:** 1 Institute of Immunology and Immunotherapy, College of Medical and Dental Science, University of Birmingham, Edgbaston, Birmingham, United Kingdom.; 2 University Hospitals Birmingham NHS Foundation Trust, Birmingham, United Kingdom.; 3 Institute of Cancer and Genomic Science, College of Medical and Dental Science, University of Birmingham, Edgbaston, Birmingham, United Kingdom.; 4 Cancer Biology Department, NCI, Cairo University, Cairo, Egypt.

## Abstract

Tumor-specific MHC class II (tsMHC-II) expression impacts tumor microenvironmental immunity. tsMHC-II positive cancer cells may act as surrogate antigen-presenting cells and targets for CD4^+^ T cell–mediated lysis. In colorectal cancer, tsMHC-II negativity is common, in cell lines due to *CIITA* promoter methylation. To clarify mechanisms of tsMHC-II repression in colorectal cancer, we analyzed colorectal cancer organoids which are epigenetically faithful to tissue of origin. 15 primary colorectal cancer organoids were treated with IFNγ ± epigenetic modifiers: flow cytometry was used for tsMHC-II expression. qRT-PCR, total RNA sequencing, nanopore sequencing, bisulfite conversion/pyrosequencing, and Western blotting was used to quantitate CIITA, STAT1, IRF1, and JAK1 expression, mutations and promoter methylation and chromatin immunoprecipitation to quantitate H3K9ac, H3K9Me2, and EZH2 occupancy at *CIITA*.

We define three types of response to IFNγ in colorectal cancer: strong, weak, and noninducibility. Delayed and restricted expression even with prolonged IFNγ exposure was due to IFNγ-mediated EZH2 occupancy at *CIITA.* tsMHC-II expression was enhanced by EZH2 and histone deacetylase inhibition in the weakly inducible organoids. Noninducibility is seen in three consensus molecular subtype 1 (CMS1) organoids due to JAK1 mutation. No organoid demonstrates CIITA promoter methylation.

Providing IFNγ signaling is intact, most colorectal cancer organoids are class II inducible. Upregulation of tsMHC-II through targeted epigenetic therapy is seen in one of fifteen organoids. Our approach can serve as a blueprint for investigating the heterogeneity of specific epigenetic mechanisms of immune suppression across individual patients in other cancers and how these might be targeted to inform the conduct of future trials of epigenetic therapies as immune adjuvants more strategically in cancer.

**Significance:**

Cancer cell expression of MHC class II significantly impacts tumor microenvironmental immunity. Previous studies investigating mechanisms of repression of IFNγ-inducible class II expression using cell lines demonstrate epigenetic silencing of IFN pathway genes as a frequent immune evasion strategy. Unlike cell lines, patient-derived organoids maintain epigenetic fidelity to tissue of origin. In the first such study, we analyze patterns, dynamics, and epigenetic control of IFNγ-induced class II expression in a series of colorectal cancer organoids.

## Introduction

The absence of tumor-specific MHC class II expression (tsMHC-II) is seen in around 50% of proficient mismatch repair (pMMR) colorectal cancer and just under 30% of deficient mismatch repair (dMMR) colorectal cancer and is associated with poor prognosis (1). Most epithelial cells do not constitutively express class II but can be induced to do so by IFNγ, which transcriptionally activates promoter IV of the class II master transcriptional activator (CIITApIV). Multiple studies have demonstrated that many cancer cell lines are not class II inducible by IFNγ due to transcriptional silencing of *CIITA* (2–5). In cell lines, this involves *CIITA* promoter CpG methylation with accompanying deficient histone H3 acetylation and high H3K9 methylation upon IFNγ treatment (2).

tsMHC-II has a profound impact on anticancer immunity. *CIITA*-transfected cancer cells are readily rejected unlike their class II negative counterparts and massively infiltrated by immune effector cells (6–8). Rejection is eliminated in IFNγ knockout mice. CD4^+^ T-cell depletion reduces the number of activated CD8^+^ T cells and abrogates the protective effect of cancer cell class II expression. CD4^+^ T-cell help for CD8^+^ T cells is crucial in the generation of robust effector and memory CTL responses (9): immunologic protection of *CIITA* transfected cells is due to the generation of primed CD4^+^ T cells providing help for the generation of cancer antigen-specific CTL effectors (7, 10). Importantly, *CIITA* transfected cells function as surrogate antigen-presenting cells (APC) priming naïve T cells in the absence of dendritic cells (DC) and other APCs (8). Unmanipulated colonic epithelial cells can take up antigen, present this to antigen-specific CD4^+^ T cells and activate them in a class II–restricted manner (11, 12). Surrogate APCs have the theoretical advantage over DCs in that they can present endogenously processed class II mutated neoantigens alongside endocytosed antigen from dying cancer cells (13).

Transfection studies involve cells with forced high-level expression, but importantly IFNγ-induced tsMHC-II also drives enhanced immunity. A recent study compared two orthotopic immunocompetent mouse models of lung cancer, one sensitive to anti-PD-1 (CMT167) and one resistant (LLC). MHC-II is not constitutively expressed on CMT167 cells but can be induced by IFNγ but cannot be induced by IFNγ on LLC cells. *In vivo* CIITA knockdown in CMT167 significantly reduces microenvironmental Th1 immunity, CD8^+^ cell number, and reduced efficacy of anti-PD-1 therapy (14). In bladder cancer, CD4^+^ tumor-infiltrating lymphocytes are directly cytotoxic for autologous cancer cells and CD4^+^ T effectors recognize antigen on cancer cells in a class II-dependent fashion (15). Intratumoral CD4^+^ T cells correlate with anti-PD-L1 efficacy. Direct CD4^+^ T cell–mediated cytolysis of melanoma cells has been described previously (16).

Given this proimmune impact of tsMHC-II expression, one of the immunologic escape mechanisms that colorectal cancer cells might utilize is cell-autonomous transcriptional repression of *CIITA* and consequent noninducibility of tsMHC-II by IFNγ. Absence of tsMHC-II may be particularly relevant in colorectal cancer due to the low abundance of conventional type 1 dendritic cells (cDC1) (17), in part related to Wnt signaling (18), which reduces intratumoral recruitment of cDC1 (19). Mice with class II negative cDC1 show weak expansion of antigen-specific CD8^+^ cells and fail to reject tumors, indicating that class II on cDC1 (the paradigmatic professional APC) is required to mediate tumor regression (20).

These studies suggest that strategies to augment tsMHC-II inducibility could improve the impact of checkpoint blockade in colorectal cancer. Given the aforementioned cell line data, the addition of DNA methyltransferase (DNMT) inhibitors would appear to be a reasonable approach, particularly in tsMHC-II negative colorectal cancer and given that DNMT inhibition alone can reverse the inhibitory histone marks of deactylated histone H3 and methylated H3K9 (21). However, the use of cell lines to investigate the epigenetic status of genes, such as *CIITA*, is problematic. CpG island hypermethylation is significantly increased in cancer cell lines compared with corresponding primary tissues (22), an effect particularly observed in colorectal cancer (23). Organoids are self-organizing three-dimensional (3D) structures that better model *in vivo* morphologies and cell-cell contacts. Importantly, over prolonged periods in culture (up to three months), intestinal epithelial organoids stably maintain the specific promoter methylation profile of the gut region from which they are derived, with recent data importantly demonstrating fidelity of methylation patterns specifically in colorectal cancer organoids (24, 25). Organoids are thus a more appropriate model to interrogate patterns of DNA methylation in colorectal cancer.

We present an analysis of tsMHC-II inducibility in a series of 15 colorectal cancer organoids, focusing on the cancer cell-autonomous mechanisms restricting class II expression via *in vitro* IFNγ stimulation. Although there are other triggers of tsMHC-II induction, IFNγ is of particular importance. The sensing by DCs of IFNγ produced by tumor-infiltrating T cells is critical to the response to anti-PD-1. IFNγ blockade abrogates tumor control by anti-PD-1 and alongside the resulting DC-produced IL12, underlies effective immune checkpoint blockade (26). The rejection of CIITA-transfected cells is eliminated in IFNγ knockout mice (7). In addition, our approach allows us to map the *in vitro* results to the microenvironmental characteristics of the cancer tissue from which the organoids are derived, something which is not possible with commercially obtained cell lines. The principal aim of this study is to understand the role, if any, of epigenetic mechanisms in silencing inducible tsMHC-II in colorectal cancer and thus guide potential therapeutic exploitation utilizing epigenetic modifiers. Such an approach, in combination with immune checkpoint blockade, could harness colorectal cancer cells as surrogate APCs and generate cancer cells as direct targets for CD4^+^ T cells and improve the effectiveness of immunotherapy in tumors such as pMMR colorectal cancer.

## Materials and Methods

### Organoid, Two-dimensional Cell Culture, and IHC

All patients were recruited at University Hospitals Birmingham NHS Foundation Trust. Patients were formally consented and ethical approval was obtained (North West-Haydock Research Ethics Committee Ref 15/NW/0079, subapproval 17-287). Organoids were derived from representative primary tumor samples. Protocols as originally described by the Hubrecht Organoid Technology (HUB)/Clevers lab were followed (27, 28). Samples were washed and broken down with tissue processing scissors in tumor digestion buffer (28), filtered and suspended in Matrigel (Corning). Intesticult Human Organoid growth media (Stemcell Technologies) supplemented with Primocin antimicrobial (Invivogen) was added to wells and plates maintained in a humidified incubator at 37°C with 5% CO_2_. Rho kinase inhibitors (Y-27632; Stratech) were used during initial derivation and freeze/thawing steps. For passaging and experimental use, organoids were separated from Matrigel using organoid harvesting solution (Bio-Techne) and a combination of mechanical and chemical digestion with TrypLE Express (Gibco). Three organoid models (COLO151, COLO155, and COLO312) were derived by the Wellcome Trust Sanger Institute as part of the Human Cancer Models Initiative Programme.

Two-dimensional (2D) cell lines including HCT116 (Horizon Discovery, RRID:CVCL_0291), RKO (AMS Biotechnology Europe Ltd, RRID:CVCL_0504), HT29 (ATCC, RRID:CVCL_0320), and DLD1 (ATCC, RRID:CVCL_0248) were obtained directly from the cell banks (supplier confirmed *Mycoplasma* and short tandem repeat integrity) as listed above. Lines used for experiments were fresh from supply/resuscitation (< 3 months and < 10 passages) and were grown in dedicated 2D culture facilities and media conditions as described routinely. Cell lines and organoids were regularly tested (every 1–2 months) for the presence of *Mycoplasma* infection with the EZ-PCR Mycoplasma Test Kit (Biological Industries). Details of previously described 2D cell line class II inducibility and CIITApIV methylation status (2) and characteristics of lines (29) summarized in Supplementary Table S1.

Tumor formalin-fixed paraffin-embedded sections were sent for Immunoscore (IS) performed by HalioDx. Primary tumor IHC staining for class II was performed using an anti-HLA DR,DP,DQ antibody (CR3/43, ab17101; Abcam, RRID:AB_443647) which has been previously validated for use in a clinical trial (ANICCA-Class II). At least two sections from each tumor were sent for blind scoring by a consultant pathologist.

### IFN and Drug Experiments

IFN stimulation was performed on whole organoid cultures, 3 days after plating to allow organoid formation. IFNγ was purchased from Stratech Scientific. A dose of 75 IU/mL was selected on the basis of maximal pathway activation (IRF1/STAT1 expression on Western blot analysis) and no incremental increase in class II positive cells (flow cytometry) observed with higher doses. GSK126 (direct selective EZH2 inhibitor) and entinostat [histone deacetylase (HDAC) inhibitor] were purchased from Stratech Scientific and 5-Azacytidine (DNA methyltransferase inhibitor) from Generon. Cell viability readouts for organoids were performed using the CellTiter-Glo 3D Cell Viability Assay (Promega) and controlled using an ATP standard curve.

### Western Blots

Cells were lysed in RIPA buffer (Thermo Fisher Scientific) with 1x Protease/Phosphatase inhibitor cocktail (Cell Signaling Technology). Quantified protein was loaded onto Mini-PROTEAN TGX Precast Gels (Bio-Rad) and resolved using SDS-PAGE and wet transfer onto polyvinylidene difluoride membranes. Membranes were probed with the following primary antibodies (see also dilutions, catalog number and where available clone numbers): STAT1 (1:1,000, #9175, 42H3, Cell Signaling Technology/CST, RRID:AB_2197984), IRF1 (1:1,000, #GTX129134, Source Bioscience, RRID:AB_2885905), Jak1 (1:1,000, #29261, E3A6M, CST, RRID:AB_2798972), GAPDH (1:1,000, #5174, D16H11, CST, RRID:AB_10622025). Blots were developed using chemiluminescence with digital capture of images performed using a Fusion FX6XT digital imaging system (Vilber Lourmat).

### Flow Cytometry

**FIGURE 1:**
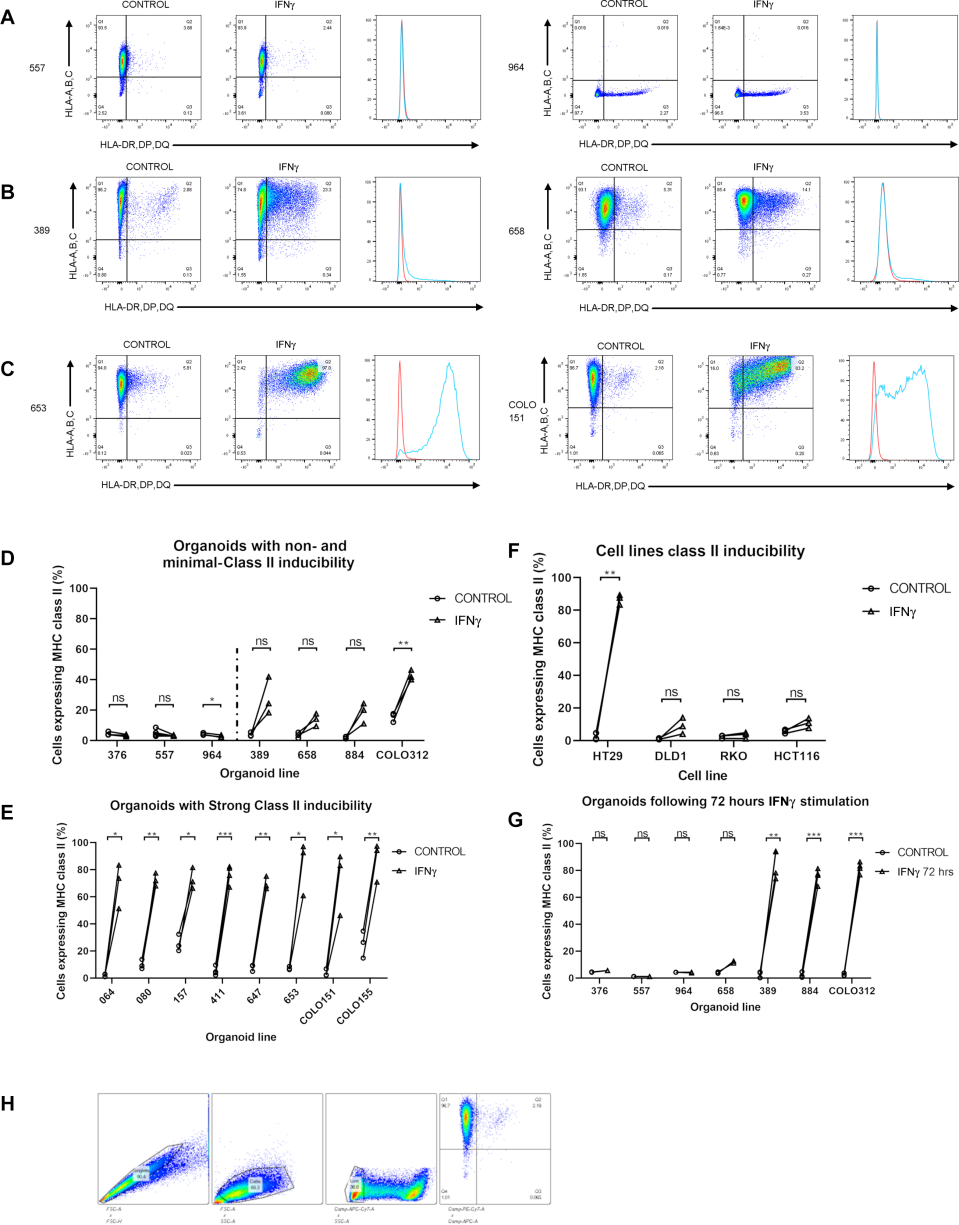
Class II inducibility across cell line and colorectal cancer organoids following IFNγ stimulation. **A**–**C,** Flow cytometry assessment of class I and II expression across lines reveal three groups of response to stimulation at 24 hours. Representative plots, with two lines displayed for each group, for noninducible (<10% class II expression; A), weakly inducible (10%–49%; B), and strongly inducible lines (>50%; C). Histogram overlay, right, for class II expression between control (red) and IFNγ (blue). Flow data from all 15 organoids available in Supplementary Fig. S1. **D–F,** Class II expression following 24 hours stimulation for all lines, performed in minimum of triplicate, unpaired *t* test (NS, *P* ≥ 0.05; *, *P* < 0.05; **, *P* < 0.01; ***, *P* < 0.001). Lines grouped into non/weakly inducible (D), strongly inducible (E), and cell lines (F). **G,** A total of 72-hour stimulation of weakly and noninducible lines, results in triplicate and statisitcs as above. **H,** Flow cytometry gating strategy.

Organoids were dissociated into single cells. Staining with eBioscience Fixable Viability Dye eFluor 780 (Thermo Fisher Scientific) followed by fluorophore-conjugated antibodies was performed. The following antibodies and dilutions were used: HLA-A,B,C (1:100, APC-conjugated, W6/32, BioLegend, RRID:AB_314879), HLA-DR,DP,DQ (3:100, PE-Cy7-conjugated, Tü39, BioLegend, RRID:AB_2564279). Gating for singlets, cells and live cells was performed and fluorescence minus one controls were used for class II, with gating strategy displayed in Fig. 1. Each experiment was performed in minimum of triplicate with comparison of stimulated and control cells under each condition. Cells were analyzed using a LSR Fortessa X-20 flow cytometer (BD), with data analysis performed using FlowJo (Version 10.5.3, BD). Compensation of data was performed in FlowJo with single-stained UltraComp eBeads (Thermo Fisher Scientific).

### DNA/RNA Isolation and *CIITA* qRT-PCR, Total RNA Sequencing, Whole-genome Sequencing, and Nanopore Sequencing

DNA and RNA were isolated from cultures using the AllPrep DNA/RNA kit (QIAGEN). DNAse treatment of RNA was performed utilizing the TURBO DNA-free kit (Thermo Fisher Scientific), followed by synthesis of cDNA using the High-Capacity RNA-to-cDNA kit (Thermo Fisher Scientific). *CIITA* and housekeeping (*GAPDH*) mRNA expression was assessed using a TaqMan fast-cycling protocol on a Quantstudio 5 using Thermo Fisher Scientific TaqMan Gene Expression arrays for *CIITA* (Hs00172106_m1) and *GAPDH* (Hs02786624_g1). Reaction was performed in quadruple for each gene and ± IFNγ, with relative increase in *CIITA* expression calculated using the 2^−(ΔΔCt)^ method.

Total RNA sequencing (RNA-seq) was performed after ribodepletion with the NEBNext Ultra II Directional RNA Library Prep Kit for Illumina (New England BioLabs). Libraries were pooled before running on the Illumina NextSeq500 platform with 75 bp paired-end reads. The normalized read count data for all expressed coding transcripts was processed by DESeq2 (v.4.0.2) software. A cutoff of gene expression fold change of ≥ 2 or ≤ 0.5 and an FDR *q* ≤ 0.05 was applied to select the most differentially expressed genes. Intrinsic consensus molecular subtype assignment was performed using the R package CMScaller (30). Gene set enrichment analysis (GSEA), Gene Ontology pathway analysis, and Kyoto Encyclopedia of Genes and Genomes pathway analysis were performed using the GAGE (RRID:SCR_017067), clusterProfiler (v3.12, RRID:SCR_016884), and pathview (v3.12, RRID:SCR_002732) packages (31–33).

Whole-genome sequencing (WGS) of DNA from organoids underwent short read WGS (MGISeq) to a depth of 60x, outsourced to Nonacus Limited. Reads were filtered and aligned to the GRCh38 genome and then underwent tumor:normal subtraction using Strelka2. Variant calls were annotated with VEP (v92, RRID:SCR_007931) and filtered to exclude any variant with a minor allele frequency (MAF) < 0.01. A Boxplot was constructed for mutations from a merged MAF file using maftools (34).

Nanopore long read sequencing including methylation calling was performed with library preparation of 1 μg of DNA using the ONT LSK-109 kit (Oxford Nanopore Technologies) according to manufacturer's protocol, and then loaded on an ONT Promethion R9.4.1 flowcell and run for 72 hours. FAST5 signal data were demultiplexed with Guppy 6 (RRID:SCR_023196), FASTQ merged, trimmed with NanoFilt (RRID:SCR_016966) and aligned to the GRCh38 reference genome using MiniMap2 (RRID:SCR_018550). Variant calling was performed with PEPPER-DeepVariant and Clair 3 using custom parameters to account for heterogenous cancer organoid samples. Structural variants were called using consensus overlaps from cuteSV and Sniffles (RRID:SCR_017619). Copy-number calling was performed using QDNASeq (RRID:SCR_003174) with 100 kb bins and methylation calling was performed using Megalodon with a 5-methylcytosine model and output to BEDGraph files.

### Bisulfite Conversion and Pyrosequencing

DNA underwent bisulfite conversion utilizing the EZ DNA methylation kit (Zymo). The sequence for the CpG island preceding CIITApIV (chr16:10,972,877-10,973,234 from UCSC; available from https://genome.ucsc.edu/) was input into the PyroMark Assay Design software (QIAGEN, version 2.0.2.5) and the following primers were obtained from Sigma-Aldrich: *CIITA* Forward: 5′-GGGGGATGGAATATGTAAAATGTAG-3′, *CIITA* Reverse-Biotin: 5′-CCCCCAAACTCTAAACACAACAAACC-3′ and *CIITA* Sequencing: 5′-GGAATATGTAAAATGTAGGG-3′. PCR amplification of the region of interest was performed on bisulfite converted DNA using the PyroMark PCR kit (QIAGEN). Finally, pyrosequencing was performed in triplicate on a PyroMark Q48 sequencer using PyroMark Q48 Advanced Reagents (QIAGEN). Analysis of C/T ratio was performed by the PyroMark AutoPrep software.

### Chromatin Immunoprecipitation

DNA was isolated from cells following treatment with 24 hours IFNγ stimulation or vehicle control. Single cross-linking of DNA with formaldehyde was performed for H3K9me2 and H3K9ac, with dual cross-linking (DSG/formaldehyde) performed for EZH2. Sonication and shearing of DNA was performed using a Covaris E220 Evolution and according to the truChIP Chromatin Shearing Kit (Covaris) protocol. Overnight immunoprecipitation was performed with the following antibodies (Abcam): H3K9ac (#ab4441, RRID:AB_2118292), H3K9me2 (#ab195482), and EZH2 (#ab195409). DNA was reversed cross-linked using Proteinase K (Roche) prior to purification with AMPure XP beads (Beckman Coulter). Four PCR primers were used to cover the whole of CIITApIV as described previously (2). PCR was performed using the PowerUp SYBR Green Master Mix (Thermo Fisher Scientific) and related protocol, with an Applied Biosystems 7500 qPCR machine and determination of *C*_t_ values and melt-curve analysis performed using SDS software (Applied Biosystems). Quantification was performed relative to a genomic DNA standard curve and data from each region was normalized by input control.

### The Cancer Genome Atlas Analysis

Normalized Agilent microarray z-score data for EZH2 and all class II genes were extracted from The Cancer Genome Atlas (TCGA, RRID:SCR_003193) colorectal dataset using the cBioportal tool (RRID:SCR_014555; refs. 35–37). Data were tabulated in Excel (Microsoft Corp.). Normality of distributions was confirmed with the Anderson–Darling test. Mean expression of all class II genes was calculated. Pearson coefficient of determination (*R* and *R*^2^) values were calculated in Excel and Minitab (Minitab Inc, RRID:SCR_014483) to investigate correlations in gene expression.

### Statistical Analysis

Statistical tests utilized are detailed alongside results and above, including unpaired *t* test, Fisher exact, and one-way ANOVA with *post hoc* test (Tukey or Sidak) performed in GraphPad Prism (version 9.0.0, RRID:SCR_002798) and other analysis, for example, Wilcoxon rank-sum in Stata 16.1 (Statcorp, RRID:SCR_012763) and Benjamini–Hochberg correction using R 4.1.0 (R Core Team, 2022, RRID:SCR_001905).

### Data Availability

The data generated in this study are available within the article and its Supplementary Data, with further raw data available upon request from the corresponding author. The genomic data have been deposited with links to BioProject accession number PRJNA978372 in the NCBI BioProject database (https://www.ncbi.nlm.nih.gov/bioproject/).

## Results

Organoids were derived from 15 primary colorectal cancers. Seven were tsMHC-II negative by tissue IHC (six of which were pMMR) and eight tsMHC-II positive (Table 1). Previous studies demonstrated that a proportion of colorectal cancer cell lines cannot be induced to express class II upon IFNγ treatment (2). We first asked whether tsMHC-II negativity is due to a cell-autonomous lack of class II inducibility in response to IFNγ. Organoids and four cell lines with previously documented *CIITA* promoter methylation status (HT29, DLD1, RKO, and HCT116; ref. 2) were treated with IFNγ and subsequent class II expression analyzed by flow cytometry. Three types of response to 24 hours IFNγ stimulation were observed: strongly inducible (>50% cells expressing class II), weakly inducible (10%–49%) and noninducible (<10%; Fig. 1A–F; Supplementary Fig. S1).

**TABLE 1:** Clinicopathologic characteristics of organoids and MHC expression

	Negative			Weak					Strong						
Organoid line	376	557	964	389	658	884	COLO312	064	080	157	411	647	653	COLO151	COLO155
Class II primary IHC (%)	0	1	0	0	20	0	0	25	0	45	1.5	75	60	0	7.5
24 hour flow class II (% cells)	3.32	3.23	2.67	28.2	13.7	18.4	42.7	69.4	72.5	73.0	76.6	69.8	78.9	72.9	87.5
72 hour flow class II (% cells)	5.54	1.31	0.21	85.1	11.9	75.9	82.0	n/a	n/a	n/a	n/a	n/a	n/a	n/a	n/a
Class I	±	+	−	+	+	+	+	+	+	−	+	−	+	+	+
Immunoscore	n/a	2	2	0	n/a	0	n/a	3	2	4	2	n/a	2	2	2
Immunoscore group	n/a	Int	Int	Low	n/a	Low	n/a	High	Int	High	Int	n/a	Int	Int	Int
CMS	1	1	1	2	n/a	2	1	2	2	1	3	4	4	3	4
Sex	M	F	M	M	F	M	F	M	F	F	F	M	F	M	M
Age at diagnosis	51	75	53	81	59	74	n/a	79	85	84	74	45	55	71	60
Left/Right sided primary	L	R	R	R	R	L	R	L	L	R	R	R	L	R	L
Stage	II	II	I	III	II	IV	II	III	II	III	IV	III	I	III	II
Grade	Poor	Poor	Mod	Mod	Poor	Poor	Poor	Mod	Mod	Poor	Mod	Poor	Mod	Mod	Mod
RAS	n/a	KRAS	n/a	—	—	NRAS	KRAS	—	n/a	n/a	—	KRAS	KRAS	—	KRAS
BRAF	n/a	—	n/a	—	V600	—	—	—	n/a	—	V600	—	—	V600	—
MMR	pMMR	dMMR	pMMR	pMMR	dMMR	pMMR	dMMR	pMMR	pMMR	dMMR	pMMR	dMMR	pMMR	pMMR	pMMR

NOTE: Organoids grouped by mean 24 hour and 72 hour class II expression as assessed by flow cytometry following IFNγ stimulation. MHC class I status (flow cytometry) as present (+), weak (±) or absent (−). Relevant clinical correlates including mean class II expression (percentage positive cells) performed by IHC on primary tumor sections, Immunoscore (for 11 samples, four technical fails by provider) including score and group (Low, Intermediate, High), consensus molecular subtype (CMS; as determined by CMScaller, one line “indeterminant”), sex, age, side of primary, tumor–node–metastasis stage and break-down, grade and extended RAS, BRAF and mismatch repair status (proficient pMMR vs. deficient dMMR) as determined by clinical pathology service.

Eight organoids demonstrated strong induction of class II expression by 24 hours (Table 1; Fig. 1C and E), including two organoids derived from tsMHC-II negative cancers, assessed by class II IHC on the primary tumor slides. In four organoids (Fig. 1B and D; Table 1), there was an intermediate pattern of class II inducibility with discernible but nonsignificant increases in class II expression at 24 hours of between 10% and 49% (weak subtype). Three of these weakly inducible organoids were tsMHC-II negative. In some fully class II inducible immortalized cell lines, full induction requires longer exposure to IFNγ (38). We therefore exposed these four organoids to 72 hours of IFNγ. This resulted in high-level class II induction in three of four of these organoids (81.4%–94.9% cells class II positive: Table 1; Fig. 1G). Finally, three organoids displayed a complete absence of class II induction to IFNγ at both 24 and at 72 hours (Table 1; Fig. 1A, D, and G). All three were IHC tsMHC-II negative ≤1%.

In colorectal cancer cell lines, noninducibility has been attributed to *CIITA* promoter methylation (2). We analyzed methylation at the CIITApIV promoter using bisulphite pyrosequencing in all 15 organoids and the four control colorectal cancer cell lines. Importantly, we found no evidence of methylation in any of the organoids including the three noninducible organoids (Table 2). Two noninducible cell lines previously identified as CIITApIV methylated (RKO and HCT116) were methylated as expected (2). On the basis of the previously observed frequency (3/8) of 2D colorectal cancer cell lines demonstrating *CIITA* methylation, this result of zero of 15 organoids nonmethylated is highly significant (probability-based *P* = 0.0009; Fisher exact *P* = 0.032). Thus, *CIITA* methylation is likely to be a 2D cell culture–mediated phenomenon and does not appear to be a relevant mechanism for silencing tsMHC-II in colorectal cancer.

**TABLE 2:** Pyrosequencing results for CIITApIV methylation

	CpG Methylation % (SD)								
Line	CpG 1	CpG 2	CpG 3	CpG 4	CpG 5	CpG 6	CpG 7	CpG 8	All
HT29	3.2 (1.2)	2.6 (0.4)	5.5 (0.4)	4.9 (0.3)	5.1 (0.2)	2.4 (0.7)	3.3 (1.3)	3.2 (1.4)	3.8 (0.4)
DLD1	6.0 (1.7)	7.1 (1.6)	12.9 (0.7)	9.1 (0.5)	13.1 (0.2)	5.4 (0.9)	5.6 (0.3)	4.9 (1.0)	8.0 (0.2)
RKO	13.0 (0.7)	15.7 (1.2)	49.9 (3.3)	44.5 (1.3)	42.3 (2.1)	37.4 (0.4)	15.1 (0.6)	16.6 (0.4)	29.3 (0.8)
HCT116	59.9 (11.0)	65.0 (5.8)	96.8 (5.5)	83.8 (1.0)	74.1 (4.1)	79.8 (1.3)	100.0 (0.0)	94.7 (2.4)	81.8 (2.7)
376	2.3 (0.7)	2.5 (0.3)	1.8 (0.4)	0.9 (0.2)	1.0 (0.1)	0.8 (0.3)	1.1 (0.3)	1.1 (0.2)	1.4 (0.2)
557	3.4 (1.1)	3.0 (0.7)	3.2 (0.4)	1.8 (0.5)	2.6 (0.7)	1.9 (1.1)	2.7 (1.6)	2.5 (1.2)	2.6 (0.7)
964	3.7 (0.6)	3.5 (1.0)	4.5 (0.5)	3.1 (0.7)	6.9 (0.5)	2.8 (1.2)	3.1 (1.5)	2.3 (1.1)	3.7 (0.8)
389	2.8 (0.7)	2.9 (0.9)	3.2 (0.7)	1.7 (0.4)	2.2 (1.1)	1.6 (0.8)	2.3 (1.2)	2.6 (1.5)	2.4 (0.6)
658	3.1 (0.7)	3.2 (1.2)	6.3 (0.4)	5.5 (0.6)	12.7 (0.8)	2.9 (0.1)	3.7 (0.8)	3.5 (1.2)	5.1 (0.3)
884	3.1 (0.4)	2.4 (0.8)	2.5 (0.3)	1.4 (0.4)	1.8 (0.9)	1.8 (1.1)	2.2 (1.2)	2.2 (1.3)	2.2 (0.5)
COLO312	3.4 (0.6)	4.4 (0.7)	11.0 (0.3)	3.0 (0.4)	3.5 (0.2)	1.2 (0.3)	1.7 (0.4)	1.9 (0.3)	3.8 (0.3)
064	2.9 (0.5)	3.7 (0.7)	4.8 (0.7)	3.0 (0.4)	4.3 (0.6)	2.8 (1.2)	3.8 (1.6)	2.7 (1.3)	3.5 (0.8)
080	2.9 (0.9)	2.6 (0.6)	3.2 (0.3)	1.6 (0.3)	5.0 (0.7)	2.4 (1.4)	2.4 (1.1)	2.0 (1.2)	2.8 (0.5)
157	2.1 (1.0)	2.4 (0.7)	3.4 (0.4)	1.6 (0.4)	1.5 (0.6)	1.5 (0.9)	1.9 (1.1)	2.3 (1.5)	2.1 (0.4)
411	3.0 (0.5)	3.3 (0.4)	3.6 (0.5)	2.7 (0.1)	3.8 (1.0)	2.0 (1.3)	3.0 (1.6)	3.3 (1.1)	3.1 (0.6)
647	3.0 (0.6)	2.9 (0.7)	2.8 (1.0)	1.3 (0.4)	1.8 (0.2)	1.1 (0.0)	1.3 (0.3)	1.7 (0.5)	2.0 (0.4)
653	3.3 (0.5)	2.8 (0.6)	2.9 (0.6)	1.8 (0.9)	1.9 (1.2)	1.9 (1.4)	2.6 (1.4)	2.1 (1.5)	2.4 (1.0)
COLO151	2.6 (1.4)	2.8 (0.6)	3.3 (0.9)	1.7 (0.7)	1.8 (0.4)	1.7 (1.1)	2.3 (1.3)	2.7 (1.9)	2.4 (0.6)
COLO155	2.2 (1.0)	2.7 (0.5)	2.9 (0.8)	1.3 (0.8)	1.8 (0.9)	1.3 (0.8)	1.8 (1.0)	1.9 (1.3)	2.0 (0.6)

NOTE: Cell line and organoid (grouped by non, weak, and strong responses to IFNγ) DNA was bisulfite converted prior to PCR and determination of CpG methylation status of CIITApIV using pyrosequencing. All experiments performed in triplicate. Results displayed show the mean methylation percentage at each CpG site and SD of data. The final column is a mean value across all CpG sites in that sample.

**FIGURE 2:**
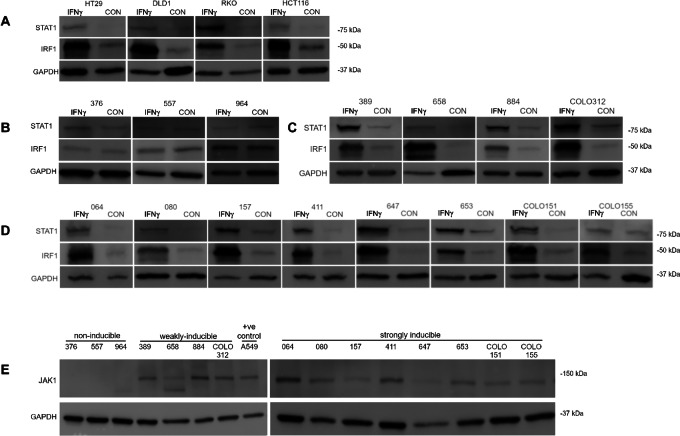
Western blot analysis for IFNγ signaling pathway component integretity. **A**–**D,** IFNγ responsiveness assessed with STAT1 and IRF1 following 24 hours stimulation demonstrating appropriate increases in protein in all cell lines (A), weakly (C), and strongly inducible (D) organoids. Loss of signaling response in noted in the noninducible lines (B). **E,** All organoid lines and A549, a control cell line with known JAK1 expression were assessed for JAK1 expression under basal conditions, with loss of protein expression confirmed in the noninducible lines. Representative blots following triplicate experiments displayed.

As the noninducible organoids had no evidence of *CIITA* methylation, we asked whether IFNγ treatment was causing appropriate activation of the proximal IFNγ pathway. Upregulation of STAT1 and IRF1 protein following IFNγ treatment was observed in all four of the control cell lines (Fig. 2A) and all of the organoids with weak or strong class II inducibility (Fig. 2C and D). However, in the three noninducible organoids, there was no change in STAT1 and IRF1 protein (Fig. 2B) suggesting a defect in proximal signaling.

**FIGURE 3:**
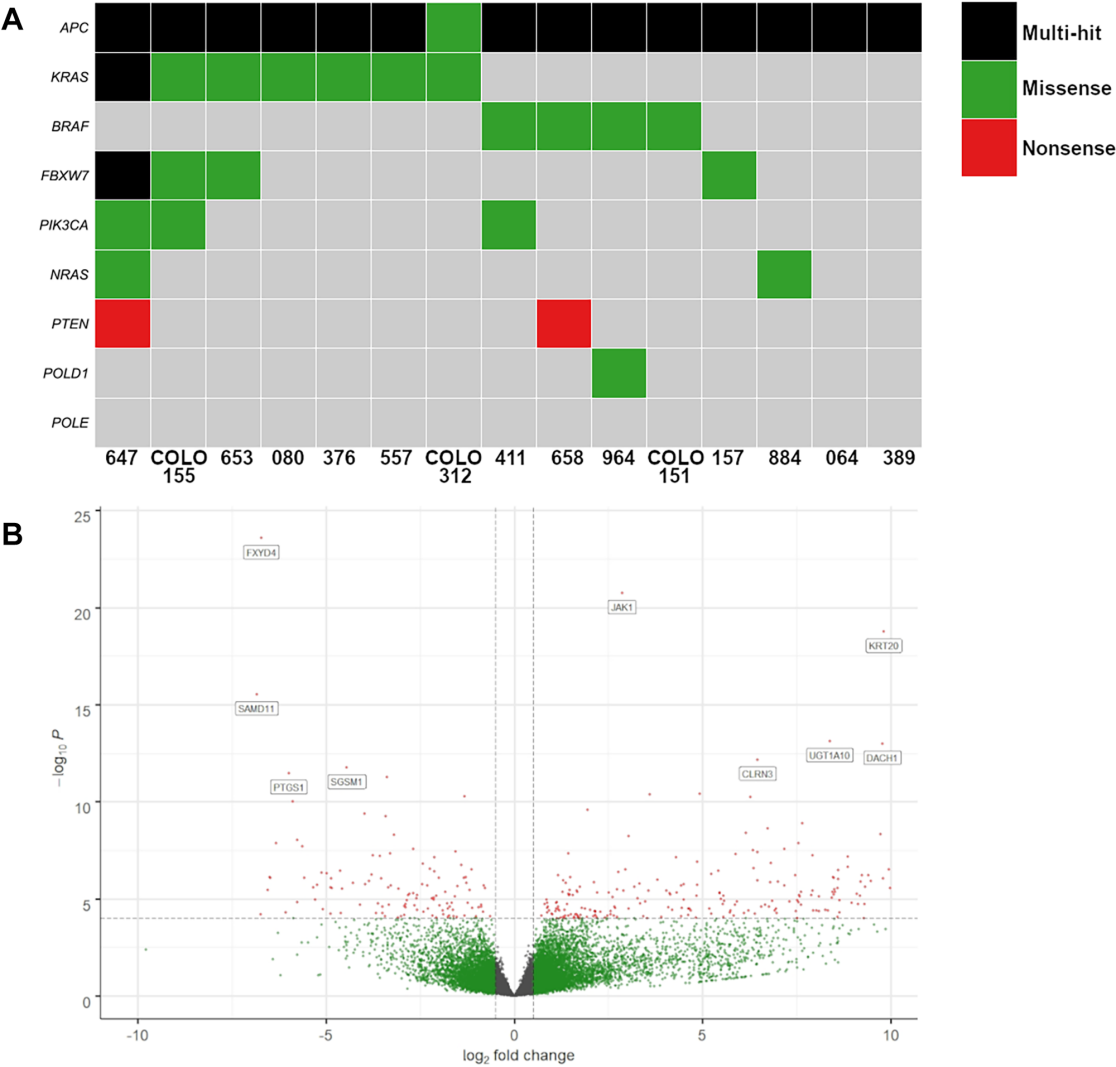
Genomic assessment of organoids. **A,** Tile plot of common mutations on WGS of organoid cultures, created with Maftools. **B,** Volcano plot for differential gene expression (RNA-seq, unstimulated organoids) between inducible and noninducible lines, most significant genes annotated including JAK1. Positive change upregulated in inducible lines.

WGS was performed on all organoids. Mutational analysis demonstrated concordance with the primary tumor pathology analysis (extended RAS and BRAF V600 clinical testing) and the observed organoid mutations (Fig. 3A; Table 1). There was no evidence of *IFNGR* mutations in any of the organoids on WGS. JAK1 is an obligatory molecule subserving downstream signaling from the IFNγ receptor and JAK1 loss is well described as an immunologic escape mechanism in dMMR colorectal cancer (39) and as a resistance mechanism to checkpoint blockade in immunogenic cancers (40). We performed RNA-seq under basal (unstimulated) conditions to see whether *JAK1, JAK2, JAK3,* or IFNγ receptor message was differentially expressed in noninducible versus inducible organoids. Following filtering, normalization and variance stabilizing transformation of all genes sequenced in this organoid dataset, 26474 genes were identified. Of these, 1,269 genes were significantly (*P* < 0.05) differentially expressed between the inducible and noninducible organoid groups (829 were upregulated in the inducible cohort and 440 were upregulated in the noninducible cohort). *JAK1* expression was the third most significant differentially expressed gene in the entire sequenced transcriptome. *JAK1* had a positive log change of 2.862, indicating higher expression in the inducible cohort (*P* = 1.21*10^−17^ Benjamini–Hochberg correction) and was the gene most differentially downregulated in the noninducible group (Fig. 3B). GSEA and Gene Ontology analysis was performed (Supplementary Fig. S2). Western blotting confirmed loss of JAK1 protein in the noninducible organoids (Fig. 2E).

Organoid consensus molecular subtype (CMS) classification recapitulates typical CMS associations (41) and all three noninducible organoids were microsatellite instability (MSI)-like consensus molecular subtype 1 (CMS1). All three contained highly deleterious mutations in the *JAK1* gene predicted to cause loss of function with two stop-gain mutations (organoid 376 *JAK1* GRCh38c.425insC p.142K/*; organoid 557 *JAK1* GRCh38c.2263C>T p.755R/*) and a 315 bp insertion into an enhancer within intron 3 of organoid 964 at GRCh38:chr1:64881273 predicted to cause loss of function. *JAK1* mutation therefore likely provides a route of immune escape in this immunogenic CMS1 cohort.

Whole-genome long-read nanopore sequencing also allows analysis of the methylation status of sequenced genes. Modified base calling of these organoids demonstrated consistent hypermethylation of the canonical *JAK1* promoter in the noninducible organoids compared with inducible (median methylation 11.8% vs. 0%, *P* = 0.0011; Supplementary Fig. S3). Although this difference is statistically significant, the degree of methylation was modest. The noninducible organoids were therefore treated with azacitidine for up to 6 days before IFNγ exposure for up to 72 hours. However, no evidence of additional class II upregulation after IFNγ (Supplementary Fig. S4) was observed, in keeping with loss-of-function mutation in *JAK1* as the main barrier to JAK1 expression in this group.

**FIGURE 4:**
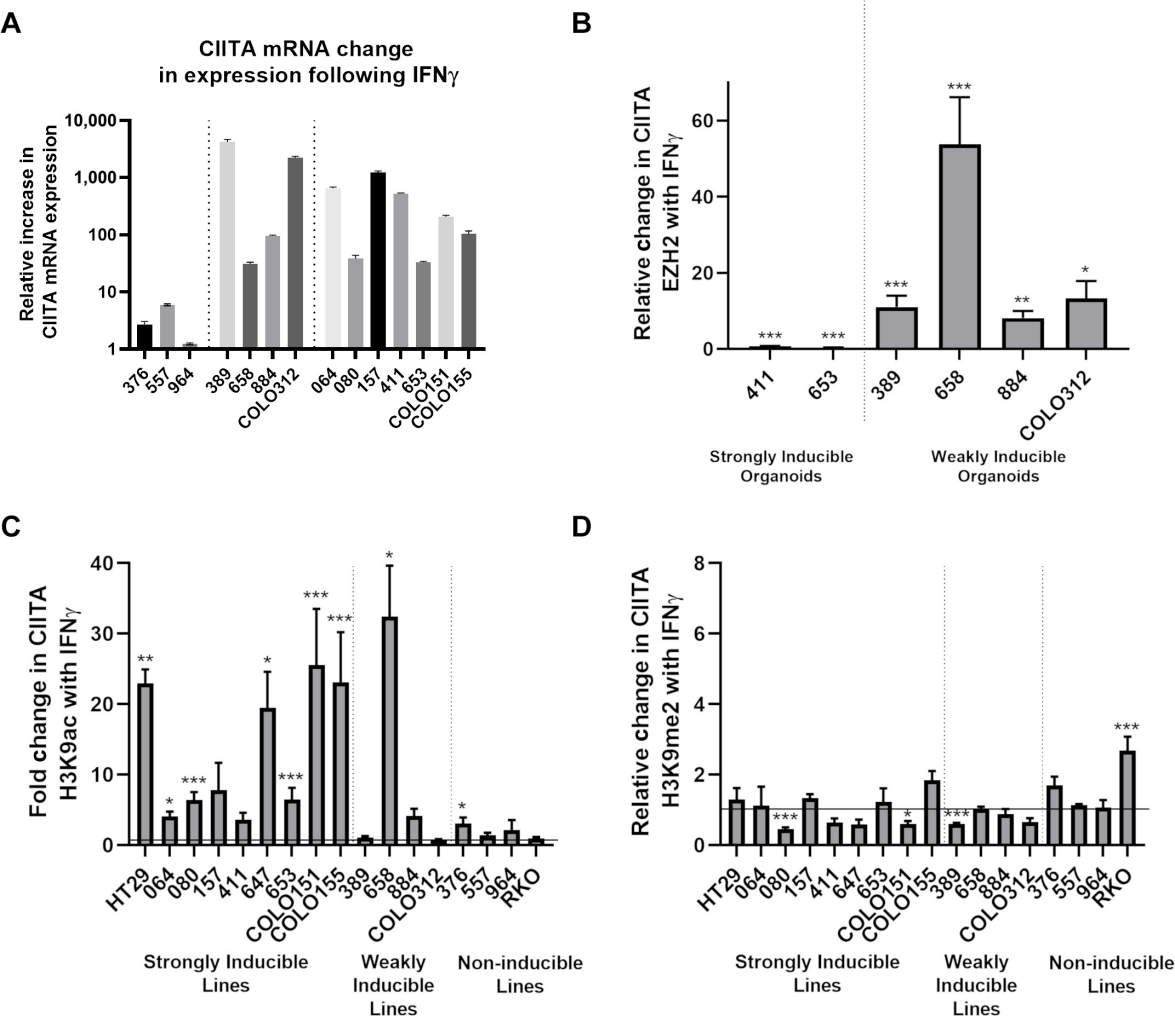
CIITA accessibility and expression following IFNγ stimulation. **A**, qRT-PCR for change in CIITA mRNA expression following stimulation across the organoid lines. Results divided by response groups (dotted lines) Calculated mean expression change and SD displayed, with four technical replicates for each line. Note logarithmic scale on *y*-axis. Statistics applied across response groups (see main text) for biological replication. **B**, ChIP PCR for EZH2 occupancy of the CIITA promoter, mean change across the four regions of the CIITA-pIV, controlled for input DNA, given for the weakly inducible lines and two representative strongly inducible lines. Results performed with four replicates, with mean and SEM plotted. ChIP PCR for change in CIITA-pIV H3K9ac (**C**) and H3K9me2 (**D**), again displaying mean across the four regions of CIITA-pIV controlled for input DNA. All organoid lines and an example inducible (HT29) and noninducible (RKO) cell line treated. Horizontal line to demonstrate no change (relative change of 1). Results performed in triplicate with mean and SEM plotted. Statsitics (unpaired *t* test) displayed for significant results *, *P* < 0.05; **, *P* < 0.01; ***, *P* < 0.001.

Of the four weakly inducible organoids, one organoid (line 658), a dMMR BRAF-mutant line, failed to significantly upregulate class II expression even with 72 hours of IFNγ (Table 1; Fig. 1G). To better understand *CIITA* accessibility after 24 hours IFNγ stimulation across this line and others, assessment of *CIITA* mRNA expression using qRT-PCR and *CIITA* occupancy/histone modification was performed using chromatin immunoprecipitation (ChIP) PCR. On qRT-PCR, the noninducible lines demonstrated the lowest relative increase of *CIITA* mRNA compared with the strong and weakly inducible organoids (Fig. 4A), with 658 having the smallest increase with IFNγ stimulation in the inducible lines. While the relative change in *CIITA* expression was similar between the weak and strong groups, the absolute levels of basal and IFNγ stimulated *CIITA* expression between the three response groups were significantly different (stimulated expression: strong vs. weak *P* = 0.0004, strong vs. negative *P* < 0.0001, weak vs. negative *P* < 0.0001, one-way ANOVA followed by Tukey *post hoc* test; Supplementary Table S2).

In highly metastatic breast cancer cell lines, an analogous subset is described demonstrating delayed class II upregulation following IFNγ exposure, mediated by IFNγ-induced EZH2 occupancy at *CIITA* (38). We analyzed EZH2 occupancy at *CIITA* using ChIP-PCR in the weakly inducible organoids and two fully inducible organoids (411 and 653) as controls. In the strongly inducible organoids, there was a decrease in EZH2-CIITApIV occupancy as expected (411: 0.73-fold reduction *P* < 0.001, 653: 0.35-fold reduction *P* < 0.001). However, in line with the breast cancer data, all four weak lines demonstrated significant increases in EZH2 occupancy at *CIITA* with 24 hours IFNγ stimulation, with a particularly large increase observed in 658 (Fig. 4B).

Dynamic changes in H3K9 acetylation (associated with chromatin accessibility) and H3K9 methylation (associated with transcriptional repression) following 24 hours IFNγ treatment were next assessed. In the strongly inducible lines and 658, there was a marked increase in *CIITA* H3K9 acetylation and no increase in H3K9 methylation (Fig. 4C and D). In the remaining three weakly inducible organoids with low-level increases in IFNγ-mediated EZH2 occupancy at 24 hours, attenuated H3K9 acetylation was observed at 24 hours. Minimal change in either H3K9 acetylation or methylation was observed in the noninducible lines, in keeping with loss of proximal pathway signaling. RKO (the methylated 2D cell line) was the only line to exhibit significantly increased H3K9 methylation following IFNγ, consistent with the promoter CpG methylation detected and as reported in the literature (2). 658 had the smallest relative induction in *CIITA* mRNA after IFNγ (Fig. 4A) of any of the inducible organoids, while still acquiring high levels of H3K9 acetylation and very high levels of EZH2 occupancy, suggesting a functional difference between this and the other organoids weakly inducible at 24 hours.

**FIGURE 5:**
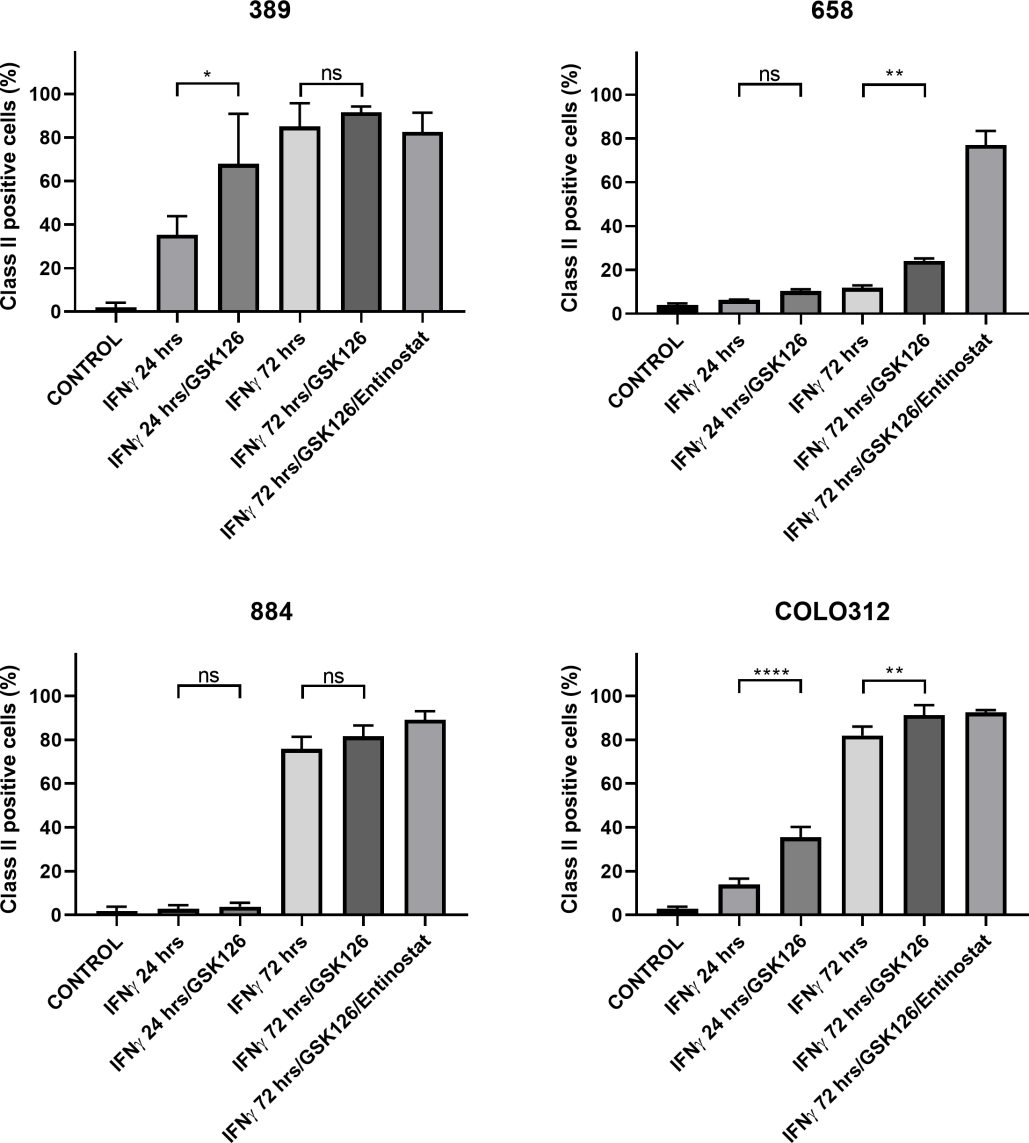
Pharmaceutical enhancement of class II inducibility in weakly inducible organoids. Weakly inducible lines were stimulated for 24 and 72 hours and additionally treated with 2 μmol/L GSK126 (EZH2 inhibitor) for 6 days ± 5 μmol/L Entinostat (HDAC inhibitor) for 72 hours. Flow cytometry data (mean class II expression and SD) displayed from experiments performed in triplicate. Statistical analysis (one-way ANOVA followed by Sidak *post hoc* test) performed for the addition of GSK126. NS, *P* ≥ 0.05; *, *P* < 0.05; **, *P* < 0.01; ***, *P* < 0.001; ****, *P* < 0.0001. The addition of Entinostat was relatively toxic with relatively few viable events and therefore statistics have not been applied.

**FIGURE 6:**
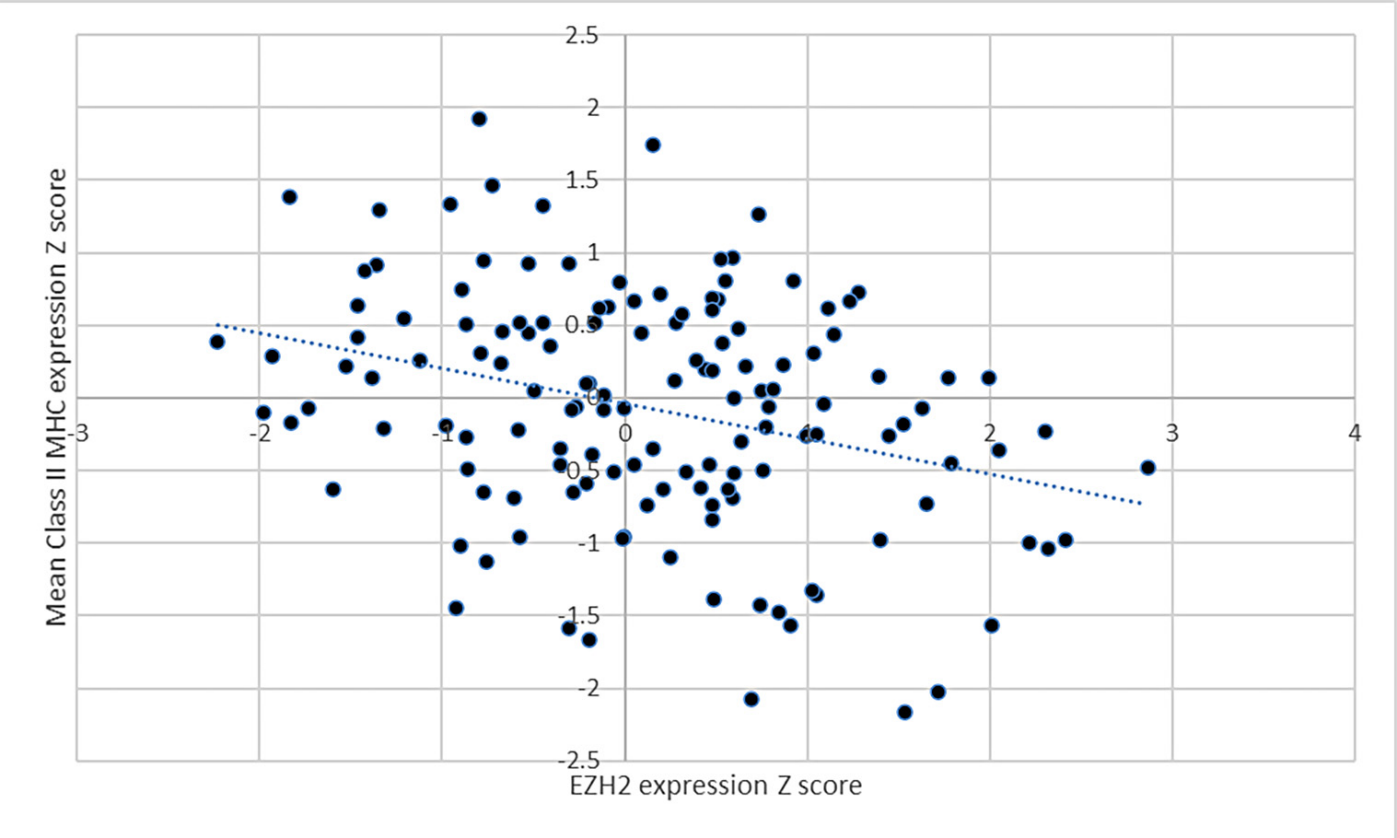
TCGA data on EZH2 and MHC class II expression. Inverse correlation (*R* = −0.314, *P* = 0.00017 Pearson correlation coefficient) of EZH2 and MHC class II expression derived from the pMMR colorectal cancer dataset. A total of 140 patients all derived from primary tumors. Characteristics: 43.6% female, 74.3% rectal/left-sided, 28.6% stage III/15% stage IV, 53.6% RAS mutant, 4.3% BRAF mutant.

We assessed the relevance of EZH2 occupancy to the reduction or delay in tsMHC-II induction. 658 was treated with the EZH2 inhibitor GSK126 plus IFNγ which increased the number of cells positive from 12.6% at 72 hours with IFNγ alone to 24.9% (Fig. 5). In the other organoids with delayed induction at 24 hours with IFNγ alone, there was also an approximate doubling of class II positive cells with GSK126/IFNγ cotreatment at 24 hours, the net effect being proportional to the amount of early induction with IFNγ alone (Fig. 5). EZH2 occupancy-mediated H3K27 methylation and H3K27 acetylation are anticorrelated marks at CIITA (38). *In vivo* HDAC1 and HDAC2 form a complex with EED/EZH2 and which has histone deacetylase activity and further EED-mediated gene repression involves histone deacetylation and can be reversed by HDAC inhibition (42). Treatment with the class I HDAC inhibitor entinostat and IFNγ increased class II expression to 65.1% of cancer cells at 72 hours in 658 and doubled the effect of GSK126 at 24 hours (Fig. 5). Finally, using TCGA data, we found an inverse correlation between EZH2 expression and mean class II expression (*R* = −0.314, *P* = 0.00017) in pMMR colorectal cancer samples (Fig. 6). Accompanying clinical characteristics provided by TCGA Network (35).

## Discussion

Using model *in vitro* systems that reproduce the promoter methylation of their tissue of origin (24, 25), we show that, providing IFNγ signaling is intact, the majority of colorectal cancer organoids are class II inducible. Furthermore, in all 15 organoids analyzed, there is no evidence of *CIITA* promoter methylation, contrary to the findings in 2D culture systems, which we have replicated herein as a positive control. There is further confirmatory evidence in colorectal cancer, with tissue microdissection from primary tissue samples failing to find any evidence of *CIITA* promoter methylation in 20 tsMHC-II negative tumors (43). Together, these findings in 3D and primary colorectal cancer samples confirm that *CIITA* methylation is not a biologically relevant route of immune evasion in colorectal cancer and is likely to be a culture-induced aberrant methylation pattern (23).

Six of eight strongly inducible organoids were tsMHC-II positive (on primary tumor IHC), two pMMR organoids, 080 and COLO312, had negative tsMHC-II on IHC. Both of these had IS = 2 suggesting that while T cells are present, they may be producing inadequate IFNγ, either because of lack of activation or functional suppression. The two other patterns of class II inducibility, absent and weakly inducible were largely associated with tsMHC-II negative cancers, with all pMMR cancers in these groups being tsMHC-II negative.

Three of 15 organoids showed loss of JAK1 expression with resultant nonresponsiveness to IFNγ secondary to *JAK1* mutations. These three organoids were all MSI-like CMS1 consistent with enhanced immunogenicity which is associated with a stronger drive to evade immune surveillance. Although the three noninducible organoids had low-level *JAK1* promoter methylation, this was not therapeutically tractable with azacitidine alone and loss of expression is much more likely to be a result of the deleterious *JAK1* mutations. JAK1 is a critical molecule linking IFNγ receptor activation to activation of all IFNγ-sensitive genes and loss of JAK1 downregulates class II, class I, and CXCL9/10 in cancer cells. In immunogenic mouse models, cancer cell JAK1 loss abrogates the effect of dual checkpoint blockade and focal irradiation and inhibits the antitumor effect of adoptive transfer of antigen-specific T cells (44). Loss-of-function mutations are well described as a cause of acquired resistance to checkpoint blockade in melanoma (40) and are seen in 17.8% dMMR colorectal cancer and 5.3% pMMR colorectal cancer (39).

Four of 15 lines were weakly inducible, however, only one of these lines (658) remained so following 72 hours IFNγ stimulation, the other three lines displaying eventual full induction with IFNγ and without the need for additional epigenetic therapies. In these four lines, high-level IFNγ-induced EZH2 occupancy at *CIITA* is observed as has previously been described, delaying and restricting class II inducibility. Sustained 72-hour IFNγ exposure in the three 72-hour inducible organoids is presumably able to overcome the much more modest increase in EZH2-CIITApIV occupancy compared with 658 (38).

We demonstrate a negative correlation between EZH2 and class II expression in colorectal cancer in TCGA dataset, in addition to the negative correlation between EZH2 and CXCL9/10 described previously (45). Our data add to the evidence supporting a key role for EZH2 in repressing MHC expression. Small cell lung cancer (SCLC) has the lowest MHC-I expression of any cancer. EZH2 is highly expressed in SCLC, and EZH2 expression is negatively correlated with both MHC-I expression and CD8^+^ T cells in clinical samples (46). EZH2 inhibition significantly reduced H32K27me3 levels and upregulated MHC-I and dramatically upregulated IFNγ-induced MHC-I expression. *In vivo* and *in vitro* EZH2 inhibition in bladder cancer significantly upregulates CIITA levels and tsMHC-II and the efficacy of therapy *in vivo* is wholly dependent on adaptive immunity. There is also an inverse correlation between EZH2 levels and immune-related transcripts in bladder cancer (47).

It is clear from our data that using epigenetic modifiers to immunologically enhance the colorectal cancer microenvironment, specifically through their positive immunomodulatory effects of enhanced tsMHC-II expression, appears to be only appropriate in a minority of cancers (those of the weakly inducible subtype). With regards to EZH2 inhibition, low concentrations of GSK126 enhance the growth of some colorectal cancer organoids (48), an effect we confirm in a subset of the organoids in this dataset (Supplementary Fig. S5). Aside from the promoting proliferation, there are additional adverse microenvironmental consequences seen with EZH2 inhibition that need to be considered carefully when utilizing these compounds clinically. Tumor-infiltrating EZH2+ T cells are polyfunctional effectors resistant to apoptosis (49). T-cell activation increases EZH2 and treatment with GSK126 decreases the proportions of polyfunctional CD8^+^ T cells and increases T-cell apoptosis. In a colorectal cancer model, GSK126 reduced the infiltration, proliferation, and IFNγ production of CD8^+^ T cells, mediated by increased myeloid-derived suppressor cells (MDSC; ref. 50).

Class I HDAC inhibitors, such as entinostat, have potent anti-MDSC effects *in vivo* (51, 52) and entinostat alone significantly upregulates IFNγ-induced class II expression in line 658 in keeping with its role in EED-mediated gene repression (42). Mocetinostat, another class I HDAC inhibitor, also significantly augments CIITA expression and class II protein expression in response to IFNγ. (53) However, as with EZH2 inhibition, HDAC inhibitors also have negative microenvironmental effects, particularly on DCs, already present at low levels in colorectal cancer (54, 55). Attempts to modulate the TME with epigenetic therapies must take into account the negative microenvironmental consequences of epigenetic therapies. Finally, while *CIITA* promoter methylation appears not to be an important mechanism of tsMHC-II loss in colorectal cancer, multiple other immune-related genes may be repressed by promoter methylation, including endogenous retroviral genes and thus the combination of DNMT inhibitors with checkpoint blockade has been studied. In the phase II trial of azacitidine plus pembrolizumab in pMMR colorectal cancer, response rate was only 3% with median progression-free survival 1.9 months despite evidence of global DNA demethylation (56). In addition, the combination of 5-azacitidine and pembrolizumab plus the addition of the HDAC inhibitor romidepsin has minimal activity in pMMR colorectal cancer (57).

A potential limitation is that the data herein was generated *in vitro*. However, we specifically wanted to explore the cell-autonomous mechanisms of class II regulation in epigenetically relevant human models. In addition, we have not examined whether upregulated tsMHC-II expression can present antigen in an efficacious manner to CD4^+^ T cells, as the remit of this work was specifically to interrogate the dynamics of class II expression itself, given the previous cell line data suggesting *CIITA*pIV methylation as an immune evasion mechanism. However, earlier data have shown that colorectal cancer cells treated with IFNγ can indeed take up, process, and present intact antigen in an HLA-DR–restricted manner (12) and immunologic protection of *CIITA* transfected is due to the generation of primed CD4^+^ T cells providing help for the generation of cancer antigen-specific cytotoxic T-cell effectors (7, 10). The relatively limited number of organoids is another limitation, but the three inducibility phenotypes described herein appear discrete and robust and we continue to derive primary colorectal organoids to elucidate whether there are further rare patterns of tsMHC-II inducibility. Furthermore, we have shown that given the number analyzed, our finding of zero of 15 organoids with methylated *CIITApIV,* compared with previous expectations from cell line data is significant and is supported by data from primary samples (43).

In conclusion, we provide an analysis of the patterns and dynamics of class II induction in colorectal cancer and the epigenetic marks that underlie them, using an *in vitro* patient-derived organoid model system and IFNγ, the crucial microenvironmental cytokine. These data demonstrate the individual variation in these dynamics and their epigenetic associations and may explain the difficulty in translating epigenetic modifiers to specifically augment tsMHC-II inducibility. This approach can serve as a blueprint for investigating the heterogeneity of specific epigenetic mechanisms of immune suppression across individual patients in other cancers and how these might be targeted to inform the conduct of future trials of epigenetic therapies as immune adjuvants more strategically in cancer.

## Supplementary Material

Supplementary Table 1 Characteristics of 2D cell lines assessed. Class II inducibility (flow cytometry) and CIITApIV methylation status as assessed by Satoh et al. Demographics, where available, from ATCC and Cellosaurus.org. Additional data on the site of tumour origin, mutation (KRAS and BRAF), mismatch repair (microsatellite instability MSI or microsatellite stable MSS) and CpG methylator phenotype (CIMP) status adapted from Ahmed et al. Full references in main text.

Supplementary Table 2 Summary of CIITA RT-qPCR results. Relative increase in CIITA mRNA expression following IFNy stimulation, mean cycle threshold (Ct) values for CIITA expression pre- and post-stimulation as determined by RT-qPCR. Each condition performed with four technical replicates. Relative expression calculated using 2^-(ΔΔCt) method and controlled using GAPDH.

Supplementary Figure 1 Full flow cytometry results from all fifteen organoid lines for Class I and Class II expression +/- IFNγ stimulation (24 hours). Histogram overlay representing change in Class II expression with stimulation with control (red) and treated (blue). See also Figure 1 a-c.

Supplementary Figure 2 RNA sequencing on unstimulated inducible vs non-inducible organoids. Gene Ontology Enrichment Analysis displayed for pathways upregulated in inducible (top) and non-inducible (bottom) organoids.

Supplementary Figure 3 Plot from Integrative Genomics Viewer (IGV) of methylation around CpG island 123 (bounded by red box) of JAK1 (GRCh38-chr1:64965773-64966931). Higher CpG methylation was demonstrated in the non-inducible (top 3 organoids 376, 557 and 964) compared with the inducible (bottom 12); 11.8% vs 0.0% (p<0.05, Wilcoxon rank-sum).

Supplementary Figure 4 Non-inducible organoids demonstrate no further upregulation of Class II following IFNγ cotreatment with Azacitidine. The three non-inducible organoids (376, 557 and 946) were treated with control or 72 hours IFNγ 75 IU/ml +/- 6 days 2 µM Azacitidine. Flow cytometry assessment of Class II upregulation from representative experiments displayed.

Supplementary Figure 5 The four weakly-inducible organoids were treated with a range of concentration of GSK126 between 0-10 µM for 72 hours, prior to determining the cell viability using CellTiter-Glo 3D Cell Viability Assay. Results were scaled relative to untreated control cells (given relative viability of 1.0) and all conditions were based on a minimum of five replicates. Mean values with standard deviation displayed. Small increases in viability noted in 3 out of 4 lines with low doses up to 2 µM, consistent with prior literature.
