# Supplementary material for: MHC Class II is Induced by IFNγ and Follows Three Distinct Patterns of Expression in Colorectal Cancer Organoids
Source: Cancer Res Commun. 2023 Aug 9;3(8):1501–13. doi: 10.1158/2767-9764.CRC-23-0091 (PMC10411481; doi:10.1158/2767-9764.CRC-23-0091)
Supplement: Supplementary Table 1 — Characteristics of 2D cell lines assessed. Class II inducibility (flow cytometry) and CIITApIV methylation status as assessed by Satoh et al. Demographics, where available, from ATCC and Cellosaurus.org. Additional data on the site of tumour origin, mutation (KRAS and BRAF), mismatch repair (microsatellite instability MSI or microsatellite stable MSS) and CpG methylator phenotype (CIMP) status adapted from Ahmed et al. Full references in main text. [file crc-23-0091-s01.docx]

| **Cell Line** | **Class II (post IFNγ)** | **CIITA-pIV methylation** | **Source** | **Demographics** | **Mutation Data** | **MSI status** | **CIMP** |
| --- | --- | --- | --- | --- | --- | --- | --- |
| RKO | Negative | Methylated | Colon primary | Unknown | *BRAF* V600E | MSI | + |
| HCT116 | Negative | Methylated | Right sided colon | 48yo M  Caucasian | *KRAS* G13D | MSI | + |
| HT29 | Positive | Unmethylated | Colon primary | 44yo F Caucasian | *BRAF* V600E | MSS | + |
| DLD1 | Positive | Unmethylated | Colon | Adult M | *KRAS* G13D | MSI | + |

**Supplementary Table 1**. **Characteristics of 2D cell lines assessed**. Class II inducibility (flow cytometry) and CIITApIV methylation status as assessed by Satoh et al. Demographics, where available, from ATCC and Cellosaurus.org. Additional data on the site of tumour origin, mutation (KRAS and BRAF), mismatch repair (microsatellite instability MSI or microsatellite stable MSS) and CpG methylator phenotype (CIMP) status adapted from Ahmed et al. Full references in main text.
