# Supplementary material for: MHC Class II is Induced by IFNγ and Follows Three Distinct Patterns of Expression in Colorectal Cancer Organoids
Source: Cancer Res Commun. 2023 Aug 9;3(8):1501–13. doi: 10.1158/2767-9764.CRC-23-0091 (PMC10411481; doi:10.1158/2767-9764.CRC-23-0091)
Supplement: Supplementary Table 2 — Summary of CIITA RT-qPCR results. Relative increase in CIITA mRNA expression following IFNy stimulation, mean cycle threshold (Ct) values for CIITA expression pre- and post-stimulation as determined by RT-qPCR. Each condition performed with four technical replicates. Relative expression calculated using 2^-(ΔΔCt) method and controlled using GAPDH. [file crc-23-0091-s02.docx]

| **Organoid** | **Relative increase in *CIITA* mRNA following IFNy** | **Mean Ct For *CIITA* (IFNy)** | **Mean Ct for *CIITA* (Control)** |
| --- | --- | --- | --- |
| 376 | 2.67 | 27.1 | 30.5 |
| 557 | 5.97 | 31.1 | 34.6 |
| 964 | 1.24 | 31.2 | 33.5 |
| 389 | 4241.7 | 23.7 | 37.5 |
| 658 | 30.9 | 28.6 | 33.5 |
| 884 | 95.8 | 24.7 | 34.5 |
| COLO312 | 2245.8 | 23.8 | 35.0 |
| 064 | 653.0 | 25.6 | 36.9 |
| 080 | 38.3 | 21.3 | 29.9 |
| 157 | 1221.5 | 22.0 | 33.2 |
| 411 | 528.8 | 24.0 | 34.0 |
| 653 | 33.3 | 21.9 | 28.5 |
| COLO151 | 206.4 | 23.2 | 31.6 |
| COLO155 | 103.9 | 22.8 | 29.8 |

**Supplementary Table 2. Summary of *CIITA* RT-qPCR results.** Relative increase in *CIITA* mRNA expression following IFNy stimulation, mean cycle threshold (Ct) values for *CIITA* expression pre- and post-stimulation as determined by RT-qPCR. Each condition performed with four technical replicates. Relative expression calculated using 2^-(ΔΔCt) method and controlled using GAPDH.
