# Supplementary material for: MHC Class II is Induced by IFNγ and Follows Three Distinct Patterns of Expression in Colorectal Cancer Organoids
Source: Cancer Res Commun. 2023 Aug 9;3(8):1501–13. doi: 10.1158/2767-9764.CRC-23-0091 (PMC10411481; doi:10.1158/2767-9764.CRC-23-0091)
Supplement: Supplementary Figure 1 — Full flow cytometry results from all fifteen organoid lines for Class I and Class II expression +/- IFNγ stimulation (24 hours). Histogram overlay representing change in Class II expression with stimulation with control (red) and treated (blue). See also Figure 1 a-c. [file crc-23-0091-s03.docx]

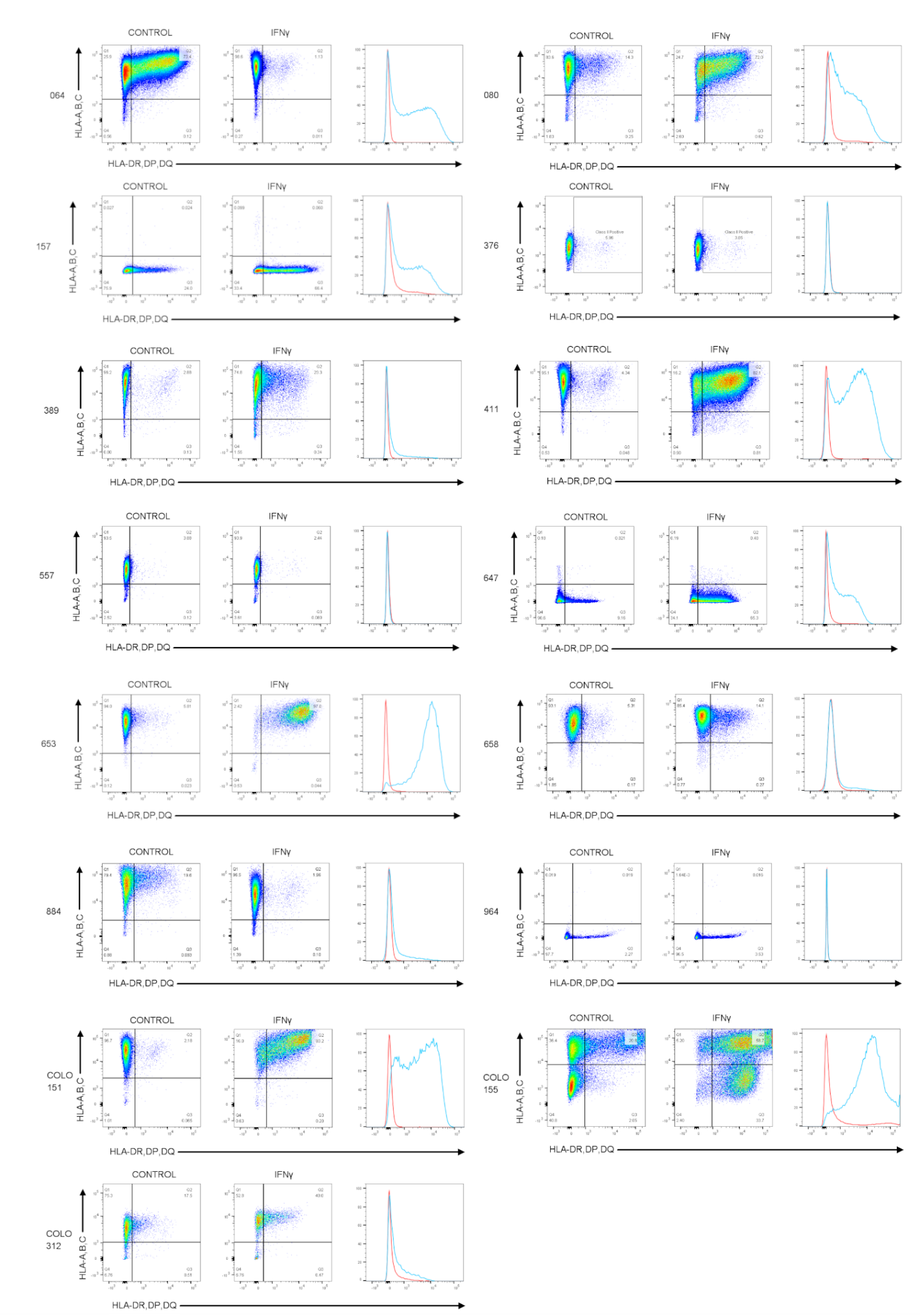


**Supplementary Figure 1** Full flow cytometry results from all fifteen organoid lines for Class I and Class II expression +/- IFNγ stimulation (24 hours). Histogram overlay representing change in Class II expression with stimulation with control (red) and treated (blue). See also Figure 1 a-c.
