# Supplementary material for: MHC Class II is Induced by IFNγ and Follows Three Distinct Patterns of Expression in Colorectal Cancer Organoids
Source: Cancer Res Commun. 2023 Aug 9;3(8):1501–13. doi: 10.1158/2767-9764.CRC-23-0091 (PMC10411481; doi:10.1158/2767-9764.CRC-23-0091)
Supplement: Supplementary Figure 2 — RNA sequencing on unstimulated inducible vs non-inducible organoids. Gene Ontology Enrichment Analysis displayed for pathways upregulated in inducible (top) and non-inducible (bottom) organoids. [file crc-23-0091-s04.docx]

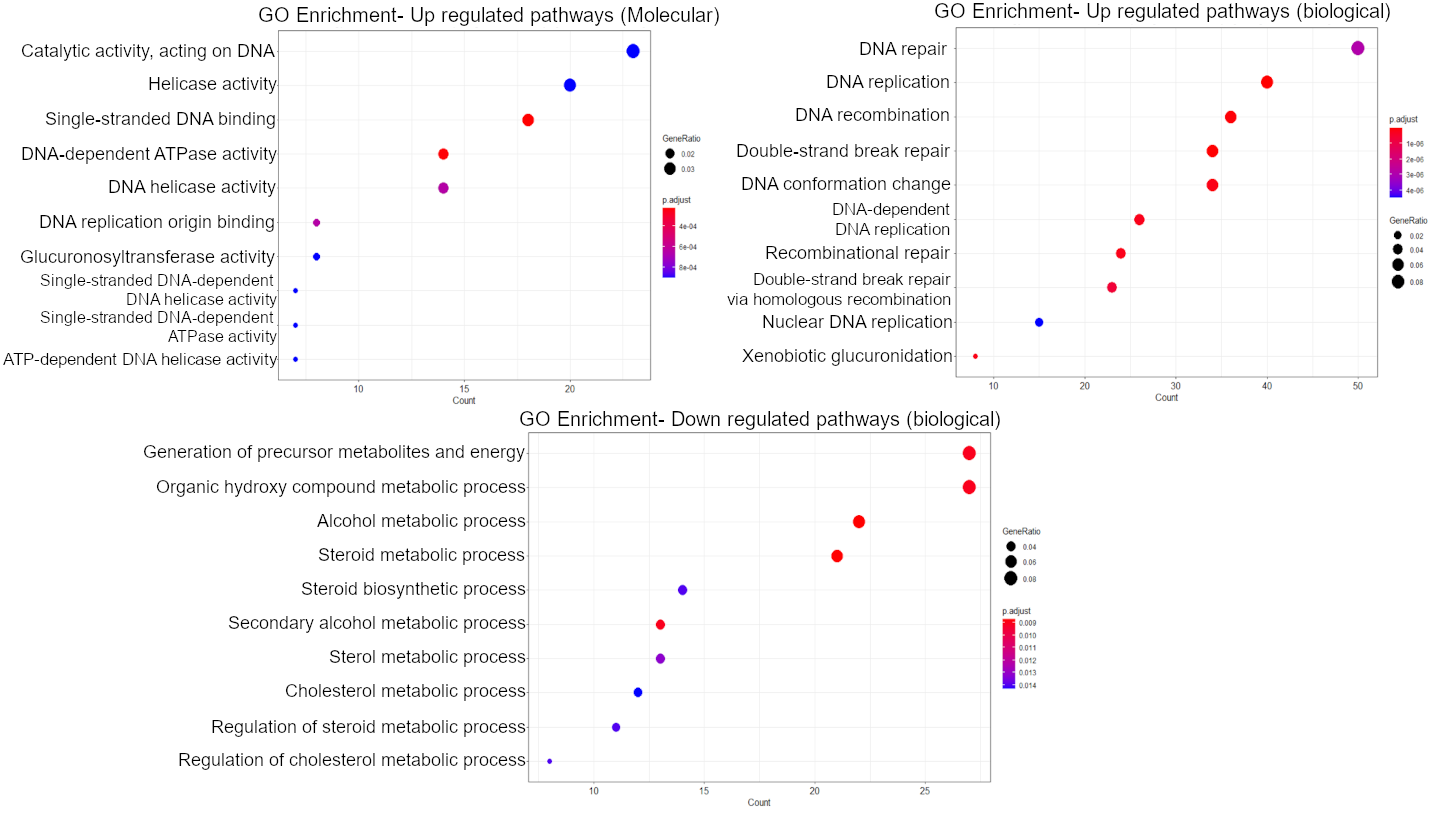


**Supplementary Figure 2** RNA sequencing on unstimulated inducible vs non-inducible organoids. Gene Ontology Enrichment Analysis displayed for pathways upregulated in inducible (top) and non-inducible (bottom) organoids.
