# Supplementary material for: MHC Class II is Induced by IFNγ and Follows Three Distinct Patterns of Expression in Colorectal Cancer Organoids
Source: Cancer Res Commun. 2023 Aug 9;3(8):1501–13. doi: 10.1158/2767-9764.CRC-23-0091 (PMC10411481; doi:10.1158/2767-9764.CRC-23-0091)
Supplement: Supplementary Figure 3 — Plot from Integrative Genomics Viewer (IGV) of methylation around CpG island 123 (bounded by red box) of JAK1 (GRCh38-chr1:64965773-64966931). Higher CpG methylation was demonstrated in the non-inducible (top 3 organoids 376, 557 and 964) compared with the inducible (bottom 12); 11.8% vs 0.0% (p<0.05, Wilcoxon rank-sum). [file crc-23-0091-s05.docx]

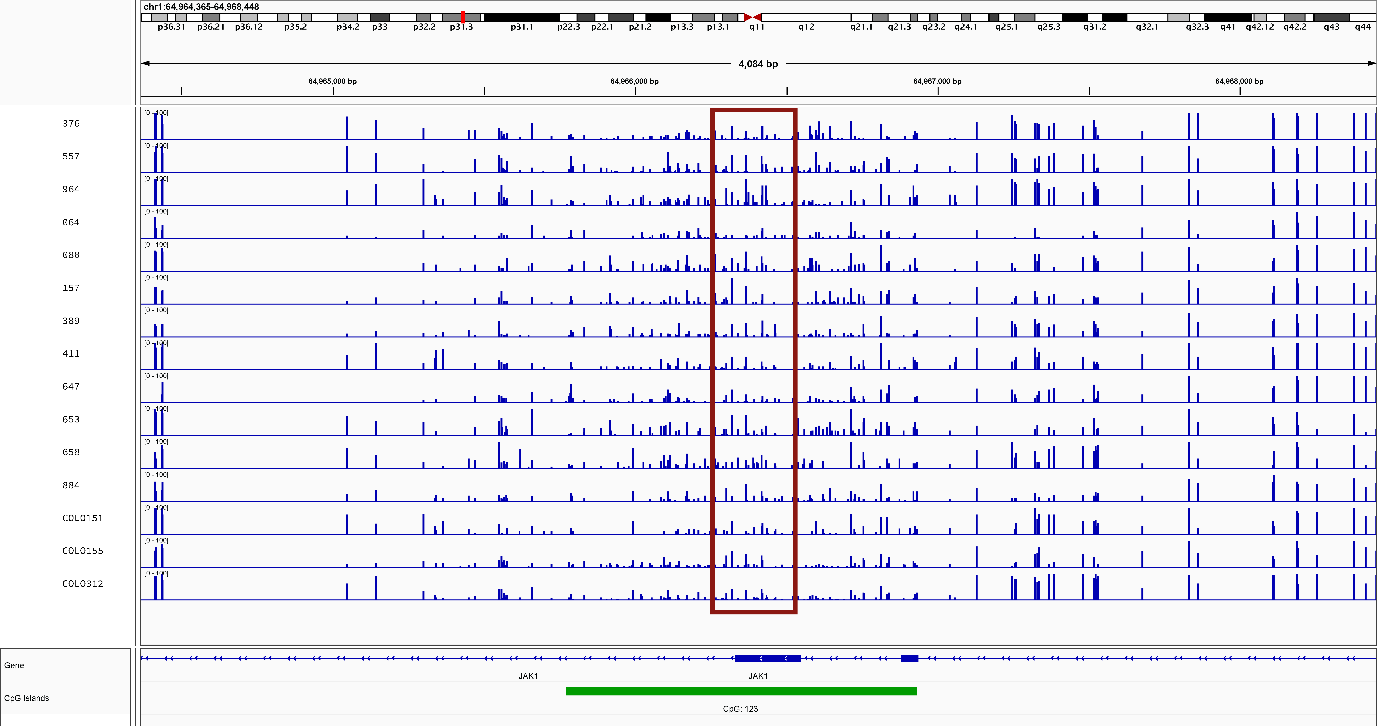


**Supplementary Figure 3 JAK1 promoter methylation** Plot from Integrative Genomics Viewer (IGV) of methylation around CpG island 123 (bounded by red box) of JAK1 (GRCh38-chr1:64965773-64966931). Higher CpG methylation was demonstrated in the non-inducible (top 3 organoids 376, 557 and 964) compared with the inducible (bottom 12); 11.8% vs 0.0% (p<0.05, Wilcoxon rank-sum).
