# Supplementary material for: MHC Class II is Induced by IFNγ and Follows Three Distinct Patterns of Expression in Colorectal Cancer Organoids
Source: Cancer Res Commun. 2023 Aug 9;3(8):1501–13. doi: 10.1158/2767-9764.CRC-23-0091 (PMC10411481; doi:10.1158/2767-9764.CRC-23-0091)
Supplement: Supplementary Figure 4 — Non-inducible organoids demonstrate no further upregulation of Class II following IFNγ cotreatment with Azacitidine. The three non-inducible organoids (376, 557 and 946) were treated with control or 72 hours IFNγ 75 IU/ml +/- 6 days 2 µM Azacitidine. Flow cytometry assessment of Class II upregulation from representative experiments displayed. [file crc-23-0091-s06.docx]

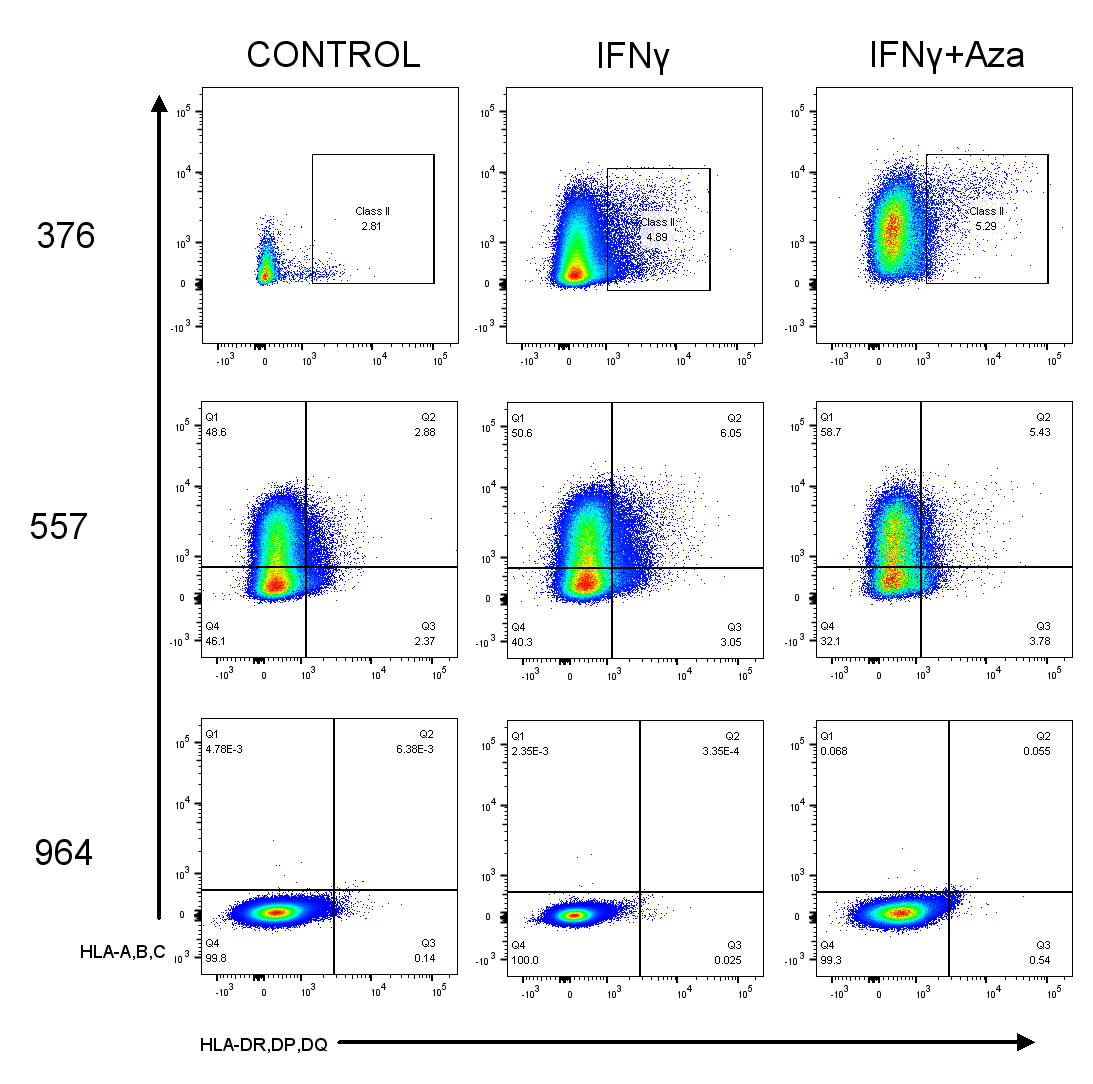
 **Supplementary Figure 4 Non-inducible organoids demonstrate no further upregulation of Class II following IFNγ cotreatment with Azacitidine.** The three non-inducible organoids (376, 557 and 946) were treated with control or 72 hours IFNγ 75 IU/ml +/- 6 days 2 µM Azacitidine. Flow cytometry assessment of Class II upregulation from representative experiments displayed.
