# Supplementary material for: MHC Class II is Induced by IFNγ and Follows Three Distinct Patterns of Expression in Colorectal Cancer Organoids
Source: Cancer Res Commun. 2023 Aug 9;3(8):1501–13. doi: 10.1158/2767-9764.CRC-23-0091 (PMC10411481; doi:10.1158/2767-9764.CRC-23-0091)
Supplement: Supplementary Figure 5 — The four weakly-inducible organoids were treated with a range of concentration of GSK126 between 0-10 µM for 72 hours, prior to determining the cell viability using CellTiter-Glo 3D Cell Viability Assay. Results were scaled relative to untreated control cells (given relative viability of 1.0) and all conditions were based on a minimum of five replicates. Mean values with standard deviation displayed. Small increases in viability noted in 3 out of 4 lines with low doses up to 2 µM, consistent with prior literature. [file crc-23-0091-s07.docx]

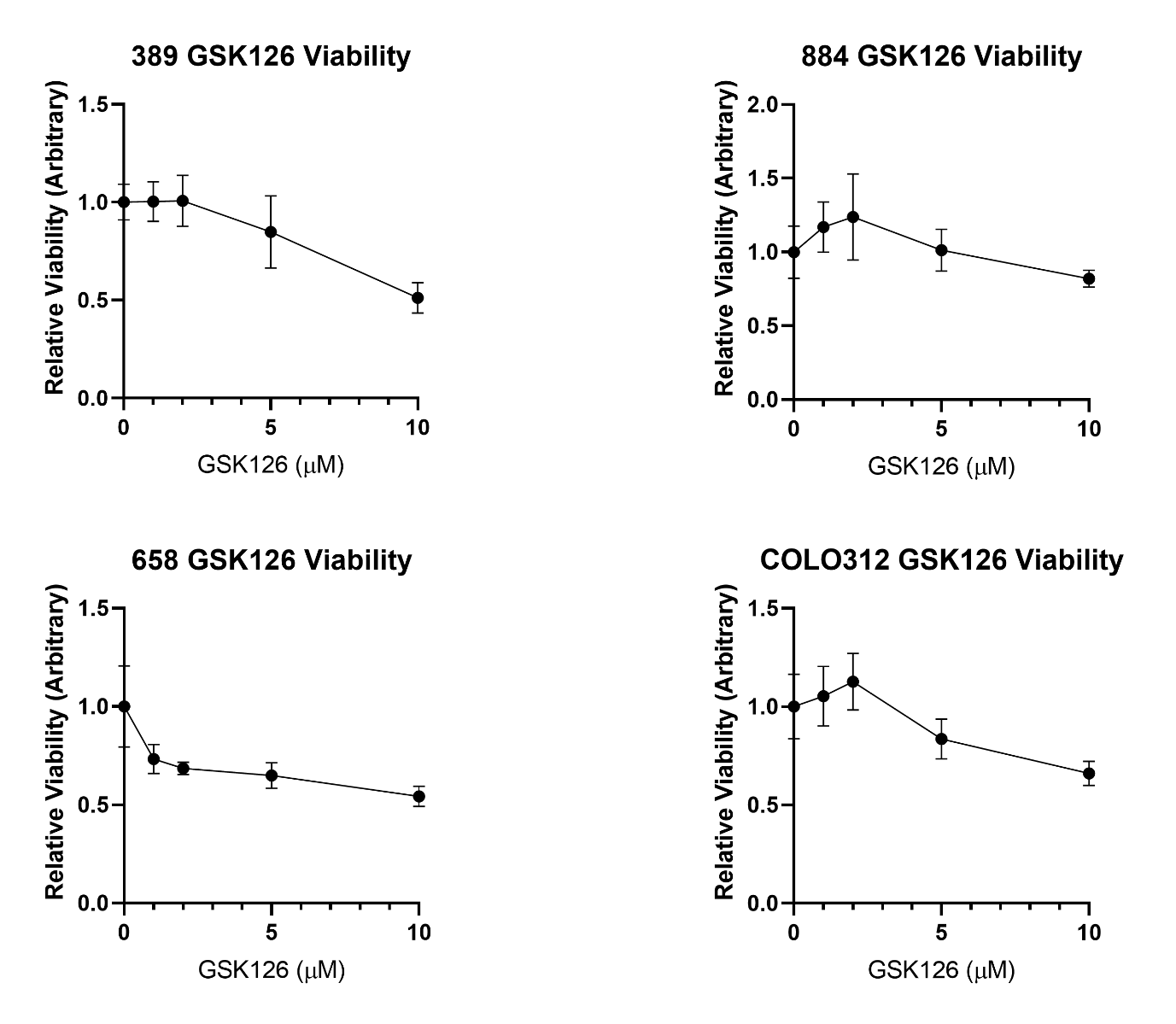


**Supplementary Figure 5 GSK126 viability across colorectal cancer organoids.** The four weakly-inducible organoids were treated with a range of concentration of GSK126 between 0-10 µM for 72 hours, prior to determining the cell viability using CellTiter-Glo 3D Cell Viability Assay. Results were scaled relative to untreated control cells (given relative viability of 1.0) and all conditions were based on a minimum of five replicates. Mean values with standard deviation displayed. Small increases in viability noted in 3 out of 4 lines with low doses up to 2 µM, consistent with prior literature.
